# Relationship between time-integrated disease activity estimated by DAS28-CRP and radiographic progression of anatomical damage in patients with early rheumatoid arthritis

**DOI:** 10.1186/1471-2474-12-120

**Published:** 2011-05-30

**Authors:** Fausto Salaffi, Marina Carotti, Alessandro Ciapetti, Stefania Gasparini, Emilio Filippucci, Walter Grassi

**Affiliations:** 1 Clinica Reumatologica, Università Politecnica delle Marche, Ancona, Italy, Ospedale "C. Urbani", Via dei Colli, 52, 60035-Jesi (Ancona)-Italy; 2 Dipartimento di Radiologia S.O.D. Radiologia Clinica, Università Politecnica delle Marche, Ancona, Italy, Ospedali Riuniti di Ancona, Via Conca 71, 60020-Torrette (Ancona)-Italy

## Abstract

**Background:**

The main aim of the study was to investigate the relationship between persistent disease activity and radiographic progression of joint damage in early rheumatoid arthritis (ERA).

**Methods:**

Forty-eight patients with active ERA was assessed every 3 months for disease activity for 3 years. Radiographic damage was measured by the Sharp/van der Heijde method (SHS). The cumulative inflammatory burden was estimated by the time-integrated values (area under the curve-AUC) of Disease Activity Score 28 joint based on C-reactive protein (DAS28-CRP) in rapid progressors versus non-progressors. Bland and Altman's 95% limits of agreement method were used to estimate the smallest detectable difference (SDD) of radiographic progression. The relationship between clinical and laboratory predictors of radiographic progression and their interactions with time was analysed by logistic regression model.

**Results:**

After 3-years of follow-up, radiographic progression was observed in 54.2% (95%CI: 39.8% to 67.5%) of patients and SDD was 9.5 for total SHS. The percentage of patients with erosive disease increased from 33.3% at baseline to 76% at 36 months. The total SHS of the progressors worsened from a median (interquartile range) of 18.5 (15-20) at baseline to 38.5 (34-42) after 3 years (p < 0.0001) whereas non-progressors worsened from a median of 14.5 (13-20) at baseline to 22.5 (20-30) after 3 years (p < 0.001). In the regression model, time-integrated values of DAS28-CRP and anti-CCP positivity have the highest positive predictive value for progression (both at level of p < 0.0001). Radiographic progression was also predicted by a positive IgM-RF (p0.0009), and a high baseline joint damage (p = 0.0044).

**Conclusions:**

These data indicate that the level of disease activity, as measured by time-integrated DAS28-CRP, anti-CCP and IgM-RF positivity and a high baseline joint damage, affects subsequent progression of radiographic damage in ERA.

## Background

Rheumatoid arthritis (RA) is a systemic chronic inflammatory disease of unknown aetiology associated with progressive joint destruction, reduction of functional capacity and quality of life and relevant social and economics costs [1-4]. Thus, early and reliable parameters for assessing the prognosis of the disease process are demanded. Radiographic joint damage is considered one of the most important outcome measures in RA with the erosive changes that appear early in the disease course, shows continuous progression and accounts for a substantial proportion of disability in RA [5-9]. Conventional plain radiography of the hands and feet is still considered the gold standard imaging technique for the assessment of joint damage progression and the effect of treatment [10-12]. Modified Sharp/van der Heijde analyses have been used in the majority of completed randomised controlled trials (RCT) [13-18]. Several studies have attempted to identify prognostic factors of radiographic progression in patients with early active RA. The main factors found are the following: socio-demographic factors (e.g. age, sex), clinical variables (disease duration, persistent swollen joint counts increased), the disease activity score (e.g. Disease Activity Score, DAS), laboratory parameters (C-reactive protein (CRP), erythrocyte sedimentation rate (ESR), high IgM rheumatoid factor (IgM-RF) titre, antibodies against citrullinated antigens (anti-CCP) and inherited factors (subtypes of HLA-DR1,-3 and-4) [19-32]. Although the relationship has been established [33-36], currently it is still difficult to predict who among the patients with early or very early RA will have radiographic progression of their disease. Such information would be important for optimizing treatment strategies.

The present analysis was performed to determine the longitudinal relationship between persistent disease activity, estimated by the time-integrated values (area under the curve-AUC) of DAS 28 joint (DAS28) based on C-reactive protein (DAS28-CRP) and subsequent radiographic progression of anatomical damage, in a cohort of patients with RA who were seen (and treated) by rheumatologists very early. We further investigated whether the longitudinal relationship between the DAS28-CRP and radiographic progression was modified by age, sex, disease duration, initial joint damage and IgM-RF or anti-CCP status at baseline.

## Methods

### Patients

Patients with early (< 1 year) active RA, attending the Rheumatology Clinic of the Università Politecnica delle Marche, Ancona, Italy and fulfilling the American College of Rheumatology (ACR) criteria [37], were included into the study and were followed for 3 years. Active disease was defined as following: ≥ 8 swollen joints, ≥ 10 tender joints and an erythrocyte sedimentation rate (ESR) of ≥ 28 mm/hour or a C-reactive protein (CRP) concentration of ≥ 1.5 mg/dl. Exclusion criteria were the following: previous used of glucocorticoids and/or disease modified antirheumatic drugs (DMARDs) within a period of three months before inclusion, alcohol abuse, serious comorbidity or recent major surgery. All patients agreed to be enrolled and provided informed consent. A cohort of 48 patients with early active RA were initially treated using a step-up approach, open to be modified during the study according to their efficacy and/or tolerance. The first DMARD used was the methotrexate (MTX). In all cases, the starting dose of oral or intramuscular MTX was 10 mg/week, increased monthly to a maximum of 20 mg/week. After 3 months, if the DAS28-CRP score remained ≥ 3.2, sulfasalazine (SSZ) was added (target dosage 40 mg/kg/day in divided doses). After the maximum tolerated dose of MTX was reached, 400 mg/day of hydroxychloroquine (HCQ) was added in patients with persistent disease activity. In all patients, MTX was co-prescribed with 5 mg/week folic acid 2 days after MTX dosing. If patients had persistent disease activity despite maximal drug therapy or drug-related toxicity, then alternative biologic agents (etanercept or adalimumab) has be used in combination in order to control disease activity. The dose of etanercept was 50 mg/week, whereas the dose of adalimumab was 40 mg every after week. Biological therapy was introduced only in 7 cases during the first year of the follow-up, in patients with a poor response to combination therapy. The use of non-steroidal anti-inflammatory drugs (NSAIDs) was allowed in all patients. The mean dosage of 6-methylprednisolone in the step-up therapy was 42.8 mg/month. Glucocorticoid therapy was tapered according to clinical judgment. At baseline all patients were monitored for medical conditions that could interfere with DMARDs therapy. The patients represent a ''real life'' sample of population with RA that can be seen at our centre. The Hospital Clinic ethics committee approved the study.

### Laboratory investigations

Baseline blood samples were obtained to evaluate the ESR (normal values ≤ 15 mm/1^st ^hour in men and ≤ 20 mm/1^st ^hour in female) and CRP (normal values ≤ 0.80 mg/dl) level, using standard laboratory methods, the presence of IgM-RF determined by nephelometric method (Image Beckman) and of anti-CCP antibodies determined by ImmunoFluoroMetric Assay (IFMA) (EliA CCP, ImmunoCAP 250, Phadia S.r.l, Italy). The cut-off point for the anti-CCP antibodies positivity was > 10 IU/ml, according to the manufacturer's instructions, whereas a titre of IgM-RF > 40 UI/ml was considered as positive.

### Clinical assessment

**Figure 1:**
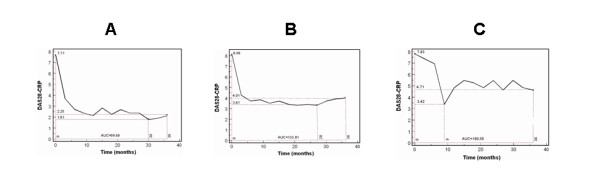
**Three representative examples showing the DAS28-CRP values in a patient with low disease activity or in remission (AUC = 99.69) (a), in a patient with moderate disease activity (AUC = 133.81) (b), and in a patient with persistently high disease activity (AUC = 180.55) (c)**.

A comprehensive questionnaire including socio-demographic data and disease-related variables was administered to the patients. At the first visit and every 3 months thereafter, the disease activity was assessed by the evaluation of the DAS28 based on C-reactive protein (CRP) concentration. The DAS-CRP combines information from the 28 tender and swollen joints, the CRP (in mg/dl) and the patient's general health status (PtGH), measured with a visual analogue scale (VAS 100 mm) [38-40]. The DAS-CRP was calculated by a WEB-site calculator http://www.das-score.nl/dasculators.html. The disease activity was interpreted as low (DAS-CRP ≤ 3.2), moderate (3.2 < DAS-CRP ≤ 5.1) or as high (DAS28 > 5.1), whilst a DAS-CRP less than 2.6 as remission, according to the European League Against Rheumatism (EULAR) criteria [41-43]. In the Figure 1 three illustrative examples of patients are reported: (a) low disease activity or remission (AUC-DAS28 = 99.69), (b) moderate persistent disease activity (AUC-DAS28 = 133.81), (c) high persistent disease activity (AUC-DAS28 = 180.55). We used the area under the disease activity curve (AUC) of the DAS28-CRP to evaluate the impact of disease activity on the progression of joint damage [44].

### Radiographic assessment

Single-emulsion radiographs of the hands and feet in anteroposterior view, were obtained and digitized at baseline and after 3 years. They were evaluated by two experienced readers, according to Sharp's method as modified by Sharp-van der Heijde Score (SHS) [45,46]. Both readers were researchers trained to score according to the SHS method and experienced in scoring radiographs in several trials [3,5,6,12,47,48]. Radiographs were scored in paired order (without information on the chronology of the films) and patient identity was blinded. For each set of radiographs, the mean score of the two readers was used for the analyses.

The SHS method assesses erosions and joint space narrowing separately and has a range from 0 to 448. Thirty-two joints in the hands and 12 in the feet were scored for erosions, with a maximum score of 5 per joint in the hands and 10 per joint in the feet. Joint space narrowing was graded from 0 to 4 in 30 joints in the hands and in 12 joints in the feet [45,46]. The principal score used in the analyses is the total score, which is the sum of the erosion score and the joint space narrowing score. Mean scores of the readers were used for the analyses. The change in the SHS, expressed as delta (∂) damage, was computed by subtracting baseline Sharp van der Heijde score values from the respective final scores. A subset of 29 chosen pairs radiographs was read twice, with an interval of at least 2 weeks in order to ascertain precision of the readings (the intraclass correlation coefficient between the two investigators was 0.91).

**Figure 2:**
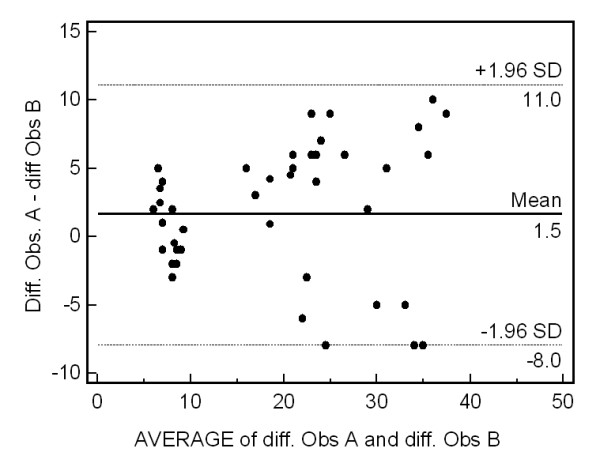
**Bland and Altman plot showing the differences in radiologic progression values plotted against average values**. Ninety-five percent of the differences against the means were less than two standard deviations (SD; dotted lines).

To determine the percentage of patients who showed a relevant radiographic change over time continuous data were dichotomised and thus a valid and clinically relevant cut-off level have been chosen. It seems logical that such a cut off value should at least be greater than the measurement error of the instrument used to quantify the response. As a starting point the smallest detectable difference (SDD) has therefore been suggested as the cut off level. The SDD is a statistical measure based on the 95% limits of agreement as described by Bland and Altman [49]. The SDD expresses the smallest difference between two independently obtained measures that can be interpreted as "real"--that is, a difference greater than the measurement error [50-53]. We thus selected all 48 pairs of radiographs of the hands and feet, with 3-year intervals, from patients with ERA. In this study, the interobserver SDD was 9.5 SHS units (2.1% of possible maximum score) (Figure 2). Using this approach, definite radiographic progression was defined as an increase in ∂ damage of 9.6 or more units between baseline and 36 months.

### Statistical analysis

Descriptive statistics are given as mean (SD) and median (interquartile range, IQR) for continuous data or as percentages for counts. The cumulative inflammatory burden was estimated by the DAS28-CRP, expressed in time-integrated values (area under the curve-AUC), [47] calculated for each patient during the 3-year follow-up. The reliability of the radiographic scores was assessed using the intraclass correlation coefficient (ICC). The ICC measures the repeatability of the scores from each reader and the repeatability of the averages of the 2 readers' scores. Wilcoxon's signed rank test was used to assess differences in SHS between baseline and 3-years follow-up. Subsequently, the variables recorded at the first visit were entered as possible explanatory variables in a regression model, using the ∂ damage between the baseline and three years as dependent variable. Covariates chosen *a priori *included the following variables: gender (as a dichotomous variable, 0 = male, 1 = female); age (as a continuous variable); disease duration (months from disease onset as a continuous variable); presence of IgM-RF (≥ 40 UI/ml) and anti-CCP antibodies (≥ 10 UI/ml); the average score of the area under the curve of the DAS28-CRP index and the baseline SHS (as a continuous variable).

Statistical analysis was performed using the Statistical Package for Social Sciences (SPSS Inc., Windows release 11.0; Chicago, Illinois, USA), and MedCalc 10.0 (Mariakerke, Belgium) statistical software.

## Results

### Patients

Fifty-nine patients were initially enrolled. Eleven patients did not complete the 3-year follow-up for the following reasons: irregular or lost follow-up (seven patients), death (one patient), transfer out (two patient), and doubts about disease duration (one patient). The final cohort included 48 patients (35 women and 13 men with mean age of 56.1 (11.1) years and mean duration of disease was 9.5 (1.9 months) whose hands and feet radiographs at 0 and 36 months were available. All patients presented high disease activity (DAS28-CRP > 5.1) at study entry. Good or moderate EULAR response criteria [41-43] were achieved by 28 patients (58.3%) at month 12, by 30 patients (62.5%) at month 24, and by 21 patients (43.7%) at month 36. A total of 11 patients (22.9%) fulfilled the EULAR remission criteria at the 3-year follow-up visit; 4 patients (8.3%) in remission at 1 year were also in remission a 3 years.

### Increase of joint destruction

The median (IQR) SHS values were 16 (13.6-20) at basal and 32 (22-39.5) 3 years after the beginning of the study. The median yearly progression score was 4.7 Sharp points (2.5-6.5). Using the SDD as the threshold level for a definite change in score ensures that the changes observed are not due to reading variability. At the end of the assessment period, the study cohort was divided into 2 groups: those whose disease was radiographically stable (subjects with a change in SHS score under the SDD) and those in whom it had progressed (subjects with a change in SHS score above the SDD). After 3-years study, 54.2% (95%CI: 39.8% to 67.5%) of RA patients (26 out of the 48) had radiographic progression greater than SDD. The percentage of erosive disease increased from 33.3% at baseline to 76% at 36 months. The median (IQR) SHS values of the rapid progressors worsened from 18 [15-20] at baseline to 38.5 [34-42] after 3 years [Δ SHS = 20.5 [18-22]] (p < 0.0001), whereas non-progressors worsened from a median of 14.5 [13-20] at baseline SHS.

### Relationship between the cumulative inflammatory burden and joint destruction

**Figure 3:**
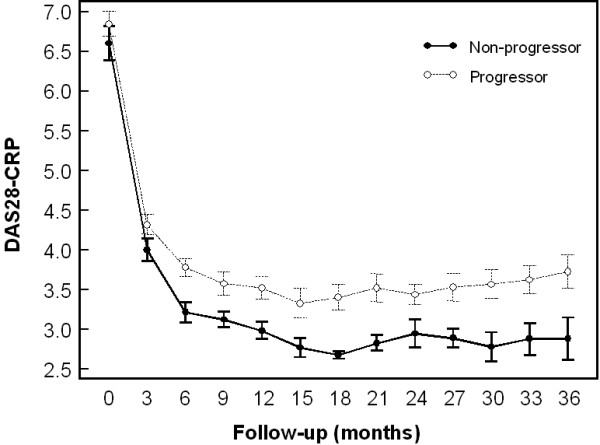
**Median scores (95% of the median) of disease activity, as measured by time integration (AUC) of the DAS28-CRP, at three-month intervals in progressor and non-progressor groups**.

The median (IQR) progression of damage at 3 years was higher (a 2.5 fold increase) and significantly different (p < 0.001) for patients showing persistent disease activity [AUC: DAS28-CRP = 130.1 (120.6-152.9)], compared with those in low disease activity or sustained remission [AUC: DAS28-CRP = 107.8 (99.7-115.5)]. Median scores (95% of the median) of disease activity, as measured by time integration at 3-month intervals in rapid progressors and non-progressors are shown in Figure 3. The radiographic progression at hand, wrist, and foot joints over a 3-year period was significantly associated with disease activity, as measured by time-integration of the DAS28-CRP (p < 0.0001), by the positivity of anti-CCP autoantibodies (p < 0.0001), and IgM-RF (p = 0.0009), and a high baseline joint damage (p = 0.0044) (Additional files 1 and 2). The age, gender and disease duration were variables not significantly associated with radiographic progression of joint damage.

## Discussion

Radiographic damage in patients with RA is one of the most important outcome measures in clinical trials and observational studies as well as in daily practice [5,7,8,10,18,48]. It is regarded as resulting from previous inflammation of the joints and is correlated with functional disability at increasing levels over time [33,34]. Other predictors include, baseline radiographic scores, the presence of IgM-RF and/or anti-CCP antibodies, specific HLADRB1 genotypes and high disease activity, disability scores and levels of acute-phase reactants [19-36,54,55]. Machold et al, [32] demonstrated that, despite early treatment, substantial damage occurred in some patients with a very early arthritis was associated with presence of strong 'constitutive' predictors such as anti-CCP antibodies and RF as well as the presence of high long-term clinical disease activity as indicated by the level of CRP, swollen joint counts and the absence of a good clinical response (assessed by the failure to achieve lasting low disease activity).

Several authors reported that baseline radiographic damage scores predict subsequent radiographic progression [56-59]. In the present study, as well as reported in literature the joint damage at baseline was a significant predictor of progression. Our prospective analysis has confirmed that the predictive accuracy of subsequent radiographic progression is greatly improved by taking into consideration the total inflammatory burden, estimated as the AUC for continuous measures of the DAS28-CRP. The AUC analysis captures two dimensions of the disease activity (magnitude and duration) in a single continuous measure [44,60]. Analysis of AUC is commonplace in other areas of medicine, for example pharmacology, quality of life research in an obvious extension. Thus, AUC of the DAS28-CRP is a very robust measure, responsive to the disease characteristics. Disease outcome is thought to be the result of the exposure to disease activity over time, rather than the result of initial disease activity [33,34].

In a cohort of active early RA patients, Knijff-Dutmer and Cohen et al [26] found a linear relationship between time integrated disease activity parameters and progression of radiographic damage was also seen. Similar results were reported by Molenaar et al [61] and Welsing et al [33]. They showed the correlation between the disease activity and the radiographic bone damage evaluated by Sharp/van der Heijde method in patients with RA follow-up for 2 years and 9 years, respectively. The hypothesis that chronic inflammation and joint destruction are closely linked is further supported by recent data from imaging studies [36,61,62] that demonstrated that in early RA bone damage occurs proportionately to the level of synovitis, but not in its absence. Using CRP for calculation of the DAS28 is an attractive alternative to ESR for a number of reasons. CRP level correlates more closely than ESR with subjective (morning stiffness, pain and fatigue after walking) and semi-objective (grip strength, articular index) and clinical parameters of RA disease activity [28,32], whereas ESR can be influenced by a number of unrelated factors, such as age, gender or plasma proteins. Laboratory tests used to calculate CRP are faster than those used to measure ESR, and measurements can be standardized in a central laboratory for multicenter clinical trials. Further, serum CRP level also has prognostic value in terms of progressive joint damage and functional status and outcome [28-30,32]. In a 3-year follow-up, van Leeuwen et al. [63] demonstrated a highly significant correlation between time integrated CRP values and radiographic progression of disease in patients with newly diagnosed RA. Plant et al. [64] prospectively examined the relationship between time-integrated CRP levels and radiographic progression in previously normal joints and already damaged joints in patients with active RA treated with DMARDs; after a 5-year follow-up period, the mean Larsen score increased from 15.9 to 36.2. Time-averaged CRP levels correlated significantly with the mean change in Larsen score over the 5-year period and a stronger correlation was seen in patients with disease duration 2 years at study entry.

Moreover, our study showed that both the presence of anti-CCP antibodies and IgM-RF correlates to the radiographic bone damage. The search for new predictive and prognostic biomarkers in patients with RA are of clinical importance [28,30,58,59] Various studies have attempered to identify prognostic factors of radiographic progression in patients with early RA. RF is one of the most powerful predictors of joint damage in early RA populations in most studies [19,26,29,32,33,65,66]. With this regard, Knijff-Dutmer et al [26] showed a correlation between a persistent disease activity evaluated by the calculation of the AUC and radiographic bone damage progression. A weak correlation between the radiographic damage progression and the presence of IgM-RF was found was also confirmed by Drossaers-Bakker *et al *[67] and by Lindqvist et al [68]. Among several autoantibodies described in recent years in patients with RA [20,23,25,28], synthetic cyclic peptides containing citrulline CCP antibodies has been proposed as a new biomarker of disease severity, since it has been found to be more sensitive than the IgM-RF by all who have published studies on this area. Positivity of anti-CCP has been found in our and in several studies, to have prognostic properties in early (and very early) arthritis, although anti-CCP antibodies may not be present at disease manifestation but may develop later in a percentage of RA patients [21-24,27]. Recent reports confirm the prognostic significance of these antibodies in early RA to be even greater than IgM-RF [69-71]. Berglin et al. reported that anti-CCP antibodies detected in preclinical phases of RA predict a poor radiographic outcome in early RA after 2 years of follow-up, whereas IgM-RF does not. Kroot et al, [70] in a study of patients with early RA found that anti-CCP positive patients at follow up had developed significantly more radiographic damage than patients without this antibody. However, in a multiple regression analysis the presence of IgM-RF was a better predictor of radiographic change (modified Sharp score) after three years than the presence of anti-CCP. Similar to our results, Bukhari et al [23] found that the presence of anti-CCP antibodies at baseline was strongly associated with both prevalent erosions (odds ratio [OR] 2.53]) and developing erosions at 5 years (OR 10.2). These ORs were higher than those for IgM-RF (OR 1.63 and 3.4, respectively).

The present study has several limitations. First, this study encompassed a relatively short period of observation, and the changes in radiographic progression seen over 3 years may not necessarily extrapolate to longer observation periods. Secondly, only a small percentage of patients received TNF-blocking agents in combination with MTX, during the follow up. Agents that block TNF, have been shown to significantly reduce joint inflammation, slow radiographic progression of joint damage, and improve physical function in clinical studies of both advanced and early RA [13,18,72,73] and this may have had some influence on the rate of the radiographic progression in this study. Further, blood tests were performed at several independent laboratories and the magnitude of intra and inter-laboratory error of ESR, CRP, anti-CCP antibodies and IgM-RF positivity has not been established and may well be significant.

## Conclusions

Estimated calculating the AUC of DAS28-CRP values obtained at 3-months interval, and the radiographic progression of joint damage at 3-year follow-up in ERA. The persistent disease activity, the presence of anti-CCP antibodies and IgM-RF at baseline and the initial joint damage were also associated with greater radiographic progression in early RA patients. This may serve for selecting patients with poor prognosis at an early stage of the disease, for more aggressive treatment [74]. Further studies on larger cohorts of patients are required to confirm our results. Our results showed a significantly positive relationship between persistent disease activity

## Competing interests

The authors declare that they have no competing interests.

## Authors' contributions

FS contributed to the conception and design of the study, to perform the data analysis and was in charge of drafting and writing the manuscript. MC contributed to the conception and design of the study, to the acquisition of data and in writing the manuscript. AC contributed to the acquisition of data and in writing the manuscript. SG contributed to the acquisition of data and to perform the data analysis. EF contributed in writing the manuscript. WG gave intellectual feedback on the manuscript. All the authors read and approved the final manuscript.

## Pre-publication history

The pre-publication history for this paper can be accessed here:

http://www.biomedcentral.com/1471-2474/12/120/prepub

## Supplementary Material

Additional file 1 **Independent predictive variables associated with radiographic progression of RA based on results of multiple regression model**. Click here for file

Additional file 2 **Scatter plots of SHS at 3 years versus with AUC-DAS28-CRP**. Click here for file
