# Supplementary material for: Relationship between time-integrated disease activity estimated by DAS28-CRP and radiographic progression of anatomical damage in patients with early rheumatoid arthritis
Source: BMC Musculoskelet Disord. 2011 May 30;12:120. doi: 10.1186/1471-2474-12-120 (PMC3123613; doi:10.1186/1471-2474-12-120)
Supplement: Additional file 1 — Independent predictive variables associated with radiographic progression of RA based on results of multiple regression model. [file 1471-2474-12-120-S1.DOC]

**Additional file 1.** Independent predictive variables associated with radiographic progression of RA based on results of multiple regression model.

| **Independent variables** | **Coefficient** | **Std. Error** | **t** | **P** |
| --- | --- | --- | --- | --- |
| *(Constant)* | -16.7337 |  |  |  |
| AUC-DAS28-CRP* | 0.1364 | 0.01853 | 7.360 | <0.0001 |
| Anti-CCP* positivity | 2.2598 | 0.5141 | 4.396 | <0.0001 |
| IgM-RF* positivity | 0.01204 | 0.003558 | 3.384 | 0.0009 |
| Joint damage at entry | 0.03366 | 0.01166 | 2.886 | 0.0044 |
| Age | 0.03750 | 0.02080 | 1.803 | 0.0732 |
| Gender | 1.2614 | 0.6496 | 1.942 | 0.0538 |
| Disease duration (months) | 0.01587 | 0.1044 | 0.152 | 0.8794 |

*AUC-DAS28-CRP = area under the curve – (AUC) of Disease Activity Score 28 (DAS28) joint based on C-reactive protein (CRP); anti-CCP = antibodies against citrullinated antigens (anti-CCP); IgM-RF = IgM rheumatoid factor (IgM-RF).
