# Supplementary figures and images for: Relationship between time-integrated disease activity estimated by DAS28-CRP and radiographic progression of anatomical damage in patients with early rheumatoid arthritis
Source: BMC Musculoskelet Disord. 2011 May 30;12:120. doi: 10.1186/1471-2474-12-120 (PMC3123613; doi:10.1186/1471-2474-12-120)

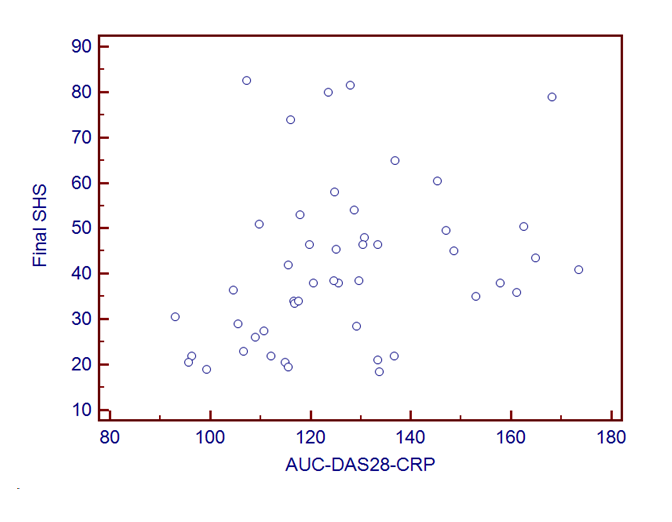

Supplement: Additional file 2 — Scatter plots of SHS at 3 years versus with AUC-DAS28-CRP. [file 1471-2474-12-120-S2.TIFF]
